# Molecular basis for METTL9-mediated N1-histidine methylation

**DOI:** 10.1038/s41421-023-00548-w

**Published:** 2023-04-04

**Authors:** Xiaoyang Wang, Huabin Xie, Qiong Guo, Dan Cao, Wenwen Ru, Shidong Zhao, Zhongliang Zhu, Jiahai Zhang, Wen Pan, Xuebiao Yao, Chao Xu

**Affiliations:** 1 MOE Key Laboratory for Membraneless Organelles and Cellular Dynamics, Hefei National Research Center for Interdisciplinary Sciences at the Microscale, Division of Life Sciences and Medicine, University of Science and Technology of China, Hefei, China; 2 Institute of Immunology, The CAS Key Laboratory of Innate Immunity and Chronic Disease, School of Basic Medical Sciences, Division of Life Sciences and Medicine, University of Science and Technology of China, Hefei, China

**Keywords:** X-ray crystallography, Post-translational modifications

Dear Editor,

Methylation is one of the most abundant and common posttranslational modifications (PTMs) and plays important roles in a wide range of cellular events^1,2^. Histidine methylation occurs at the N1 or N3 position of the imidazole ring and accounts for ~13% of protein methylation events^3^. Although methylhistidine was found in actin and myosin decades ago^4^, very few mammalian histidine-specific methyltransferases were identified until recently, and several groups identified SETD3 and METTL18 as actin and RPL3 histidine-N3 methyltransferases, respectively^5–8^, and METTL9 as a histidine-N1 methyltransferase^9,10^. Unlike SETD3 or METTL18, which methylates a unique substrate, METTL9 specifically recognizes an xHxH motif (H is for histidine and x denotes small residues.) and catalyzes the methylation of the second histidine. xHxH, as a known metal binding motif, is found in a wide range of metal binding proteins, suggesting the potential role of METTL9-dependent histidine methylation in mediating the metal binding capacities for those proteins. Despite its important role, the molecular mechanism underlying substrate recognition and methylation by METTL9 is largely unknown.

To provide mechanistic insight into the substrate binding property of METTL9, we purified different METTL9 fragments, which are easy to aggregate and have low solubility. Based on the predicted METTL9 structure from AlphaFold^11^, we chose to mutate its surface-exposed hydrophobic residues to stabilize the protein. One METTL9 fragment (METTL9^46–318^) with six mutations, named METTL9^M6^ afterwards, exhibited high solubility and homogeneity (Fig. 1a and Supplementary Fig. S1a). Then, we synthesized peptides derived from previously identified substrates, including SLC39A5^369–380^ and mouse S100A9^101–111^ ^9,10^, and examined their binding affinities for METTL9^46–318^ and METTL9^M6^ by isothermal titration calorimetry (ITC) binding assay. Both variants of METTL9 bind peptides with similar affinities (K_d_s: 2.3–9.3 μM vs 3.6–9.0 μM) (Fig. 1b, c) to that between SETD3 and actin peptide^6^. Furthermore, mass spectrometry (MS) experiments showed that both METTL9^46–318^ and METTL9^M6^ catalyze the methylation of SLC39A5^369–380^. No methylated products were found without METTL9 (Supplementary Fig. S2). Altogether, the binding assay and MS data demonstrate that both METTL9^46–318^ and METTL9^M6^ recognize and efficiently methylate peptide substrates.

**Fig. 1:**
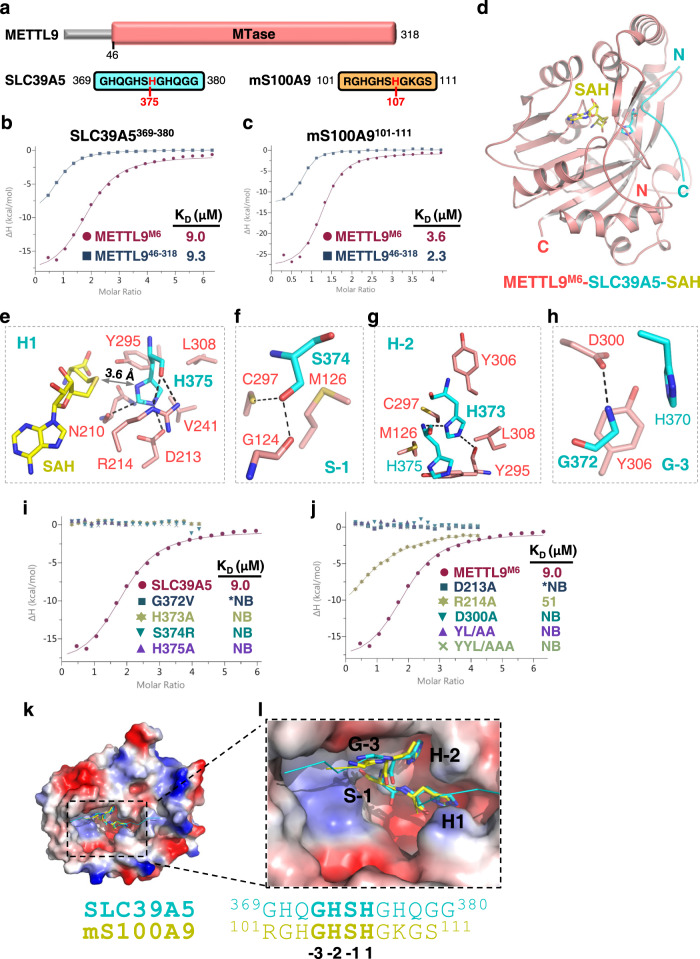
METTL9-mediated substrate recognition and catalysis. **a** Domain architecture of human METTL9 and the sequences of SLC39A5^369–380^ and mouse mS100A9^101–111^, with the substrate histidines colored in red. **b** ITC curves for SLC39A5^369–380^ bound to wild-type METTL9^46–318^ and METTL9^M6^. **c** ITC curves for mS100A9^101–111^ bound to wild-type METTL9^46–318^ and METTL9^M6^. **d** Overall structure of METTL9^M6^ bound with SAH and SLC39A5^369–380^. METTL9^M6^ and SLC39A5 peptides are shown in red and cyan cartoons, respectively, and SAH is shown in yellow sticks. **e**–**h** Sequence-specific recognition of xHxH by METTL9^M6^. Detailed interactions between METTL9^M6^ and H1 (**e**), S-1 (**f**), H-2 (**g**), and G-3 (**h**). **i** ITC curves for METTL9^M6^ binding to SLC39A5^369–380^ and its variants. **j** ITC curves for SLC39A5^369–380^ bound to METTL9^M6^ and its variants. YL/AA and YYL/AAA are for Y306A/L308A and Y295A/Y306A/L308A, respectively. **k**, **l** The structures of SLC39A5-bound METTL9^M6^ and mS100A9-bound METTL9^M6^ are superimposed, with the electrostatic surface of METTL9^M6^ shown. The superimposed GHSH motifs of SLC39A5 and mS100A9 are labeled and shown as cyan and yellow sticks, respectively.

Given that crystallization trials on the wild type failed, we used METTL9^M6^ for structural studies and obtained the crystal structures of a METTL9 variant in complex with SAH and different peptide substrates, including mouse S100A9^101–111^ and human SLC39A5^369–380^, at resolutions of 3.43 Å and 1.69 Å, respectively (Fig. 1d and Supplementary Table S1). In the 1.69 Å SLC39A5-bound structure, METTL9^M6^ adopts a canonical seven-β-strand (7BS) MTase architecture characterized by the seven-strand β4-β3-β2-β5-β6-β10-β9 β-sheet (Fig. 1d and Supplementary Fig. S1a, b). SAH is buried in a deep groove of METTL9^M6^ formed by β2, β3, β5, α7, and α8 (Supplementary Fig. S1c). All peptide residues in the SLC39A5 peptide, including the C-terminal tyrosine (C-Tyr), were well resolved in the structure (Supplementary Fig. S3a). The SLC39A5^369–380^ peptide adopts an extended conformation and lies in a deep groove of METTL9^M6^ formed by the loop between α4 and β1, the loop between β5 and α8, β9, β10, and the loop between β9 and β10 (Supplementary Fig. S1a, b). As predicted, all six mutations of METTL9^M6^ are solvent-exposed, with five of them in α3 and one between α3 and α4; that is, none of them is close to the SAH-binding site or substrate-binding site (Supplementary Fig. S1a).

SAH interacts with METTL9^M6^ mainly via hydrogen bonding interactions, with its adenosine ring sandwiched between Leu175 and Leu211 of METTL9^M6^. Specifically, the ribose O3’ and O4’ atoms of SAH are hydrogen bonded to Glu174 of METTL9^M6^; the SAH amide is hydrogen bonded to the main chain of Leu209; and the carboxylate groups of SAH form polar interactions with the side chains of Asn210 and Tyr295 (Supplementary Fig. S1d). All SAH binding residues except Leu175 are absolutely conserved across species, suggesting that METTL9 employs a conserved mode to bind SAM/SAH (Supplementary Fig. S1a).

In the METTL9–SLC39A5 interface, extensive intermolecular interactions were found between METTL9^M6^ and the ^372^GHSH^375^ motif of SLC39A5. SLC39A5 His375 (“H1”), as the substrate histidine, is accommodated into a deep pocket of METTL9^M6^ composed of Asn210, Asp213, Arg214, Val241, and Tyr295. The C2 and N3 atoms of His375 are hydrogen bonded to the main chain carbonyl group of Asn210 and the side chain carboxyl group of Asp213 of METTL9^M6^, respectively. The carbon‒oxygen hydrogen bond represents unusual hydrogen bonding interactions in biomacromolecules^12^. His375 imidazole ring stacks with Tyr295, Arg214, and Val241 of METTL9^M6^ (Fig. 1e). The distance between His375 and SAH is 3.6 Å, suggesting that the complex structure captures the premethyl transfer status (Fig. 1e). The hydrogen bond between N3 of His375 and Asp213 would facilitate the deprotonation of N1 and subsequent methyl transfer. In addition to His375, Ser374 of SLC39A5 (“S-1”) forms two hydrogen bonds with Cys297 and Gly124 of METTL9^M6^ and forms van der Waals interactions with Met126 (Fig. 1f). His373 of SLC39A5 (“H-2”) is snugly accommodated into a cleft on the METTL9^M6^ surface formed by Met126, Tyr295, Cys297, Tyr306, and Leu308, with its imidazole ring sandwiched between Cys297 and Leu308 and buttressed by Tyr295. SLC39A5 His373 also forms an intramolecular hydrogen bond with His375 and an intermolecular hydrogen bond with METTL9^M6^ Tyr295 via its N1 and N3 atoms, respectively (Fig. 1g), suggesting that methylation of His373 (H-2) at the N1 or N3 atom disrupts the binding and inhibits the methylation of His375 (H1). SLC39A5 Gly372 (“G-3”) forms a hydrogen bond with Asp300 of METTL9^M6^ via its main chain amide group and stacks with His370 of SLC39A5 and Tyr306 of METTL9^M6^ (Fig. 1h), suggesting that any bulky residue at this position would introduce steric clash. SLC39A5 His370 (“H-5”) forms additional van der Waals interactions with Tyr302 and Tyr306 of METTL9^M6^ (Supplementary Fig. S4a). In contrast to ^370^HQGHSH^375^, residues downstream of His375, ^376^GHQGG^380^, make less contact with METTL9 (Supplementary Fig. S4b).

Then, we employed structure-directed mutagenesis experiments to evaluate the roles of SLC39A5 residues in binding to METTL9^M6^. Consistent with the structural analyses, SLC39A5 single mutants, including H375A, S374R, H373A, and G372V, disrupt the binding to METTL9^M6^ (Fig. 1i), suggesting that METTL9 recognizes the xHxH motif in a sequence-specific manner. Intriguingly, the substrate His375 is also critical for substrate recognition. Next, we mutated METTL9^M6^ residues to evaluate their roles in binding to SLC39A5. While R214A weakened the SALC39A5 binding affinity by ~5-fold, single mutants D213A and D300A, double mutant Y306A/L308A, and triple mutant Y295A/Y306A/L308A abolished the binding to SLC39A5 (Fig. 1j). We further examined the circular dichroism (CD) spectra of METTL9^M6^ and the two mutants, Y306A/L308A and Y295A/Y306A/L308A. The CD spectra of the three proteins are largely similar (Supplementary Fig. S5), suggesting that the mutations do not influence the overall fold of METTL9^M6^. Collectively, mutagenesis experiments and ITC binding assays further validate the substrate-binding interface.

Next, we performed a methylation assay for METTL9^M6^ and the two mutants, E174A and Y306A/L308A, with the SLC39A5 peptide as the substrate. Glu174 is a SAM-binding residue, while Tyr306 and Tyr308 are both substrate-binding residues. The *k*_cat_/*K*_m_ value for METTL9^M6^ is 0.37 μM^−1^ h^−1^, comparable to those of other methyltransferases^13^. In contrast, neither E174A nor Y306A/L308A displayed detectable activity toward the SLC39A5 peptide (Supplementary Fig. S6). The enzyme assay suggested that both SAM- and substrate-binding residues play critical roles in METTL9^M6^-mediated histidine methylation.

The structure of METTL9^M6^–mS100A9 is largely similar to that of METTL9^M6^–SLC39A5, with residues 103–108 of the mS100A9 peptide well resolved (Supplementary Fig. S3b) and the substrate histidine His107 positioned in the catalytic pocket (Supplementary Fig. S7). METTL9^M6^ recognizes the xHxH motif in mS100A9 (^104^GHSH^107^) in the same manner as shown in the METTL9^M6^–SLC39A5 complex (Supplementary Fig. S7b). Superposition of the two substrate-bound METTL9^M6^ structures indicates that the two GHSH motifs align very well, suggesting the conserved xHxH recognition mode by METTL9^M6^ (Fig. 1k, l).

Next, we solved the 2.10 Å structure of METTL9^M6^ bound with SAH and methylated SLC39A5 peptide (Supplementary Fig. S8a and Table S1), in which the peptide is well resolved with His375 methylated at the N1 atom (Supplementary Fig. S3c), demonstrating the histidine N1-specific methylation activity of METTL9^M6^. The distance between the methyl group of His375me and SAH is 3.0 Å (Supplementary Fig. S8b), indicating that it is a postreactive structure. The product-bound structure is identical to that of the substrate-bound structure (Supplementary Fig. S8c, d), indicating that no remarkable conformational changes occur during catalysis. This finding is in contrast with our previously solved SETD3 structures, in which the imidazole ring of actin histidine rotates ~90° during catalysis^6^.

It has been reported that METTL9 prefers an Ala at the −1 position in addition to Ser^10^. To understand the selectivity of METTL9 toward residues at the −1 position, we further solved the structure of METTL9^M6^ with the SLC39A5 mutant S374A (S-1A) at a resolution of 2.75 Å (Supplementary Fig. S9a and Table S1) with the mutant peptide well resolved (Supplementary Fig. S3d). In the structure, Ala374 (“A-1”) forms hydrophobic interactions with Met126 and Cys297 of METTL9^M6^, with a distance of 3.8 Å between Ala374 and the main chain carbonyl group of METTL9^M6^ Gly124 (Supplementary Fig. S9b). Any residue at the −1 position larger than Ser or Ala is disfavored because of potential steric clash with the main chain of Gly124. In all METTL9^M6^ complexes, H1 and H-2 are snugly positioned into histidine-specific pockets, while x-1 forms hydrogen bond or hydrophobic interactions with METTL9^M6^ and x-3 stacks with the aromatic ring of METTL9^M6^ Tyr306 (Fig. 1f, h and Supplementary Fig. S9b). The above sequence-specific interactions account for the preferred recognition of xHxH by METTL9.

Previously, the structures of two histidine methyltransferases, CARNMT1^14^ and SETD3^6,15^, were solved, which prompted us to compare their structures with that of METLL9. In all three histidine methyltransferase structures, histidine-specific hydrogen bonding interactions were found between the imidazole ring of the substrate histidine and the acidic residue of the enzyme, which play structural roles in positioning the substrate histidine for catalysis and promoting histidine deprotonation for methyl transfer (Supplementary Fig. S10). In the structure of METTL9^M6^ bound with the SLC39A5 peptide, the substrate histidine (His375) is snugly fitted into the catalytic pocket. The histidine-specific interactions, including hydrogen bonding and packing interactions, position the N1 atom of His375 close to the SAM cofactor to enable N1-specific methyl transfer (Supplementary Fig. S10a). Consistent with the spacious catalytic pocket of SETD3, the His-to-Ala mutation only slightly weakened the substrate binding affinity, and the substrate histidine rotated its imidazole ring by ~90° during catalysis^6^. In contrast, H375A abolished the substrate binding affinity of METTL9 (Fig. 1i), and the side chain of the substrate histidine did not change orientation during METTL9-mediated methyl transfer, suggesting their different substrate properties.

In summary, the study unveils the mechanism underlying the N1-specific histidine methylation of the xHxH motif by human METTL9 and deepens our understanding of the chemical and enzymological properties of METTL9, which sheds light on the future design of potent inhibitors for the broad-specificity histidine-N1 methyltransferase.

## Supplementary information

Supplementary Information file

## Data Availability

The coordinates and structure factors for the structures of METTL9 were deposited into the Protein Data Bank under accession codes 7YF2, 7Y9C, 7YF3, and 7YF4.
