# Supplementary material for: Molecular basis for METTL9-mediated N1-histidine methylation
Source: Cell Discov. 2023 Apr 4;9:38. doi: 10.1038/s41421-023-00548-w (PMC10073072; doi:10.1038/s41421-023-00548-w)
Supplement: Supplementary file 1 — Supplementary Information file [file 41421_2023_548_MOESM1_ESM.pdf]

Supplementary Information

Expression and purification of METTL9^{M6} and its variants

The cDNAs encoding full length human METTL9 and different METTL9 fragments are cloned into a modified pET28a-SUMO vector. Six point mutations on METTL9$^{46-318}$ were performed using two reverse and complement primers to generate METTL9^{M6}. METTL9^{M6} recombinant protein was overexpressed in *Escherichia coli* BL21(DE3). *E. coli* cells were grown in LB medium at 37°C until the optical density (OD_{600}) reached ~0.8. Protein expression was induced with 0.2 mM β-d-1-thiogalactopyranoside (final concentration) for 20 h at 16°C. Cells were collected by centrifugation at 5000 rpm for 15 min at 4°C, and pellets were resuspended in lysis buffer containing 20 mM Tris-HCl, pH 7.5, and 400 mM NaCl. After purification by Ni-NTA (GE Healthcare), SUMO protease was added to remove the N-terminal SUMO-tag of METTL9^{M6}. After dialysis with lysis buffer overnight, the mixture was applied to another Ni-NTA resin to remove the protease and uncleaved proteins. The cleaved recombinant protein was further purified by a HiLoad 16/600 Superdex 75 column (GE healthcare) in a buffer containing 20 mM Tris-HCl, pH 8.0, 150 mM NaCl, and 1 mM EDTA. The protein solution was diluted to approximately 50 mM NaCl and further purified by HiTrap Q-HP (GE Healthcare) with a linear gradient from 50 mM to 1 M NaCl. Other METTL9 variants were purified in the same way.

Crystallization, data collection, and structure determination.

All crystals were grown using the sitting drop vapor diffusion method at 18°C. For the crystallization of METTL9^{M6} with SLC39A5, recombinant METTL9^{M6} (10 mg/mL) was preincubated with a synthesized SLC39A5 peptide (aa 369-380) and SAH (S-(5′-adenosyl)-L-homocysteine) (Sigma–Aldrich) at a molar ratio of 1:2:3. The mixture was further mixed in a 1:1 ratio (v/v) with the crystallization buffer containing 0.1 M BINCEN pH 9.0, 2% 1,4-dioxine, 10% w/v PEG 20000, 12% glycerol, and 0.01 M TCEP. For the crystallization of METTL9^{M6} with methylated SLC39A5, recombinant METTL9^{M6} (10 mg/mL) was preincubated with synthesized SLC39A5 peptide (aa 369-380) and SAM at a ratio of 1:3:2 and mixed with the crystallization buffer containing 0.1 M MES pH 8.0, 12% w/v PEG 20000. For the crystallization of METTL9^{M6} with

the SLC39A5 mutant peptide (aa 369-380: GHQGHAHGHQGG), $METTL9^{M6}$ (10 mg/mL) was preincubated with the synthesized peptide and SAH at a molar ratio of 1:3:4 and mixed with the crystallization buffer containing 0.1 MES pH 8.0, 12% w/v PEG 20000. For the crystallization of $METTL9^{M6}$ with mS100A9, $METTL9^{M6}$ (10 mg/mL) was preincubated with the synthesized mS100A9 peptide (aa 101-111) and SAH at a molar ratio of 1:4:3, and the mixture was further mixed in a 1:1 ratio with the crystallization buffer containing 0.1 M Tris pH 8.5, 0.2 M $MgCl_2$, 30% (w/v) PEG 4000, 0.1 M ND SB-256. The diffraction data were collected at 0.9791 Å on beamline BL18U1 at Shanghai Synchrotron Facility. Data sets were processed using HKL3000 [1] or XDS[2]. The crystal structures were solved by using the model from AlphaFold (https://alphafold.ebi.ac.uk/)[3] for molecular replacement with Phenix [4]. After molecular replacement, the peptides were built manually by WinCoot[5]. The structure was further refined by Phenix[4]. The statistics for data collection and refinement are summarized in supplementary table 1.

Mass spectrometry

Reversed-phase microcapillary/tandem mass spectrometry (LC/MS/MS) was performed using an Easy-nLC nanoflow HPLC (Proxeon Biosciences) with a self-packed 75 µm × 15 cm C18 column connected to a Q ExactiveTM Plus (Thermo Scientific). A 50 µL reaction mixture comprising 4 µM $METTL9^{46\text{-}318}$ or $METTL9^{M6}$, 40 µM peptide and 60 µM SAM in a buffer containing 10 mM Tris-HCl (pH 8.0) and 20 mM NaCl was incubated at 37°C for 2 h before being quenched at 70°C for 15 min. Then, the reaction was treated with a Zip Tip C18 column (Millipore) to remove NaCl before analysis by LC/MS/MS. The data were analyzed by Xcalibur software (Thermo Scientific), with the relative abundances of substrate and product, which reflect the activity of protein histidine methyltransferase.

Isothermal titration calorimetry (ITC)

Proteins were dialyzed against the ITC buffer containing 20 mM Tris pH 8.0, 150 mM NaCl). Peptides were solved in H_2O to a final concentration of 30-50 mM, and were diluted in the ITC buffer to 1 mM for each ITC trial. ITC binding experiments were carried out at 25°C on a MicroCal iTC200 calorimeter (GE Healthcare) in ITC buffer

by titrating 2 μL of peptides (1 mM) in the syringe into the cell containing 50 μM protein, with a spacing time of 120 s and a reference power of 5 μCal/s. Control experiments were performed by titrating 1 mM peptides into the ITC buffer and were subtracted during analysis. All binding experiments were performed at least in duplicate to ensure the reproducibility of the data. Binding isotherms were plotted, analyzed and fitted based on a one-site binding model by MicroCal PEAQ-ITC Analysis software (Malvern Panalytical) after subtraction of the respective controls.

Circular Dichroism (CD)

Recombinant METTL9^{M6} and its mutants, Y306A/L308A and Y295A/Y306A/L308A, were purified as described above. Circular dichroism (CD) spectra were recorded on a Circular Dichroic Spectrometer J1700 (JASCO, Japan) at room temperature in a buffer containing 10 mM KH_2PO_4 and 100 mM $(NH4)_2SO_4$ using a quartz cuvette with a cell length of 0.1 cm. Before recording, the concentration of all purified protein was adjusted to 0.2 mg/mL. The wavelength range used for measurement is 190-250 nm. Raw data were recorded and analyzed by SPECTRA MANAGER Software (JASCO, Japan) and processed using the GraphPad Prism 8 software (GraphPad Software, USA).

Steady-state kinetic measurement

The catalytic activity of METTL9 was measured by using the MTase-Glo™ Methyltransferase Assay (Promega, USA)[6]. The 25 μL of reaction mixture contains 20 mM Tris-HCl pH 7.5, 50 mM NaCl, 1 mM $MgCl_2$ and 1 mM DTT. Various concentrations of SLC39A5$^{365-380}$ (from 40 nM to 40 μM) were mixed with 40 μM of SAM.The methylation reaction was catalyzed by 2 μM recombinant METTL9^{M6} or its mutants, E179A and Y306A/L308A. After 1 hour of incubation at 37 °C, the reaction was quenched by adding 0.1% trifluoroacetic acid (TFA), and the steady-state kinetic measurement was performed to follow the instruction. Luminescence detection was performed on Synergy™ H1 microplate reader (BioTek, USA). The k_{cat} and K_m were calculated by the GraphPad prism 8 software (GraphPad Software, USA) according to the Michaelis–Menten equation.

References

1 Minor W, Cymborowski M, Otwinowski Z, Chruszcz M. HKL-3000: the integration of data reduction and structure solution--from diffraction images to an initial model in minutes. *Acta Crystallogr D Biol Crystallogr* 2006; **62**:859-866.

2 Kabsch W. Xds. *Acta Crystallogr D Biol Crystallogr* 2010; **66**:125-132.

3 Jumper J, Evans R, Pritzel A *et al.* Highly accurate protein structure prediction with AlphaFold. *Nature* 2021; **596**:583-589.

4 Adams PD, Afonine PV, Bunkoczi G *et al.* PHENIX: a comprehensive Python-based system for macromolecular structure solution. *Acta Crystallogr D Biol Crystallogr* 2010; **66**:213-221.

5 Emsley P, Cowtan K. Coot: model-building tools for molecular graphics. *Acta Crystallogr D Biol Crystallogr* 2004; **60**:2126-2132.

6 Hsiao K, Zegzouti H, Goueli SA. Methyltransferase-Glo: a universal, bioluminescent and homogenous assay for monitoring all classes of methyltransferases. *Epigenomics* 2016; **8**:321-339.

Supplementary Table S1. Data collection and refinement statistics

	METTL9[M6]-SLC39A5[369-380]	METTL9[M6]-methylated SLC39A5[369-380]	METTL9[M6]-S100A9[101-111]	METTL9[M6]-SLC39A5[369-380] S374A
**PDB ID**	**7YF2**	**7Y9C**	**7YF3**	**7YF4**
**Data collection**				
Space group	C 1 2 1	I 1 2 1	C 1 2 1	C 1 2 1
Cell dimensions				
a, *b*, *c* (Å)	143.84,45.84,106.42	106.28,46.05,124.78	141.89,43.55,106.49	142.39,43.21,106.23
α, β, γ (°)	90,122.64,90	90,103.35,90	90,123.70,90	90, 122.77,90
Resolution (Å)	60.56-1.69(1.78-1.69)[a]	43.05-2.10(2.16-2.10)	70.32-3.43 (3.64-3.43)	59.86-2.75(2.90-2.75)
$^{b}R_{merge}$	0.055(0.482)	0.081(0.424)	0.256(0.673)	0.132(0.764)
$I / \sigma I$	16(2.8)	14.6(3.2)	5.8(2.4)	9.9(2.6)
$CC_{1/2}$	0.999(0.892)	0.998(0.880)	0.984(0.797)	0.997(0.894)
Completeness (%)	97.4(89.9)	99.6(96.2)	94.7(80.8)	97.3(99.9)
Redundancy	5.6(4.5)	6.4(5.0)	6.3(5.6)	5.9(5.4)
**Refinement**				
Resolution (Å)	35.42-1.69	31.83-2.10	70.32-3.43	59.86-2.75
No. reflections	63898	34530	7067	14118
R_{work} / R_{free}	0.179/0.218	0.192/0.243	0.236/0.288	0.223/0.285
No. atoms				
Protein	4360	4319	4183	4162
Peptide	176	175	92	94
SAH	52	52	52	52
Water	456	394	N/A	7
B-factors				
Protein	32.2	28.3	60.2	60.3
Peptide	40.7	33.1	59.4	60.8
SAH	23.5	22.1	56.3	53.6
Water	38.2	30.7	N/A	53.4
R.m.s. deviations				
Bond lengths (Å)	0.009	0.007	0.002	0.005
Bond angles (°)	1.1	0.92	0.54	0.83
Ramachandran Plot				
favored/allowed/outliers (%)	98.8/1.2/0	98.0/2.0/0	96.6/3.4/0	94.7/5.3/0

[a]Values in parentheses correspond to the highest resolution shells.

$^{b}R_{merge} = \Sigma_{hkl}\Sigma_j$ | I(hkl;j)−<I(hkl)> | /(ΣhklΣj<I(hkl)>), where I(hkl;j) is the jth measurement of the intensity of the unique reflection (hkl), and I(hkl) is the mean overall symmetry-related measurements.

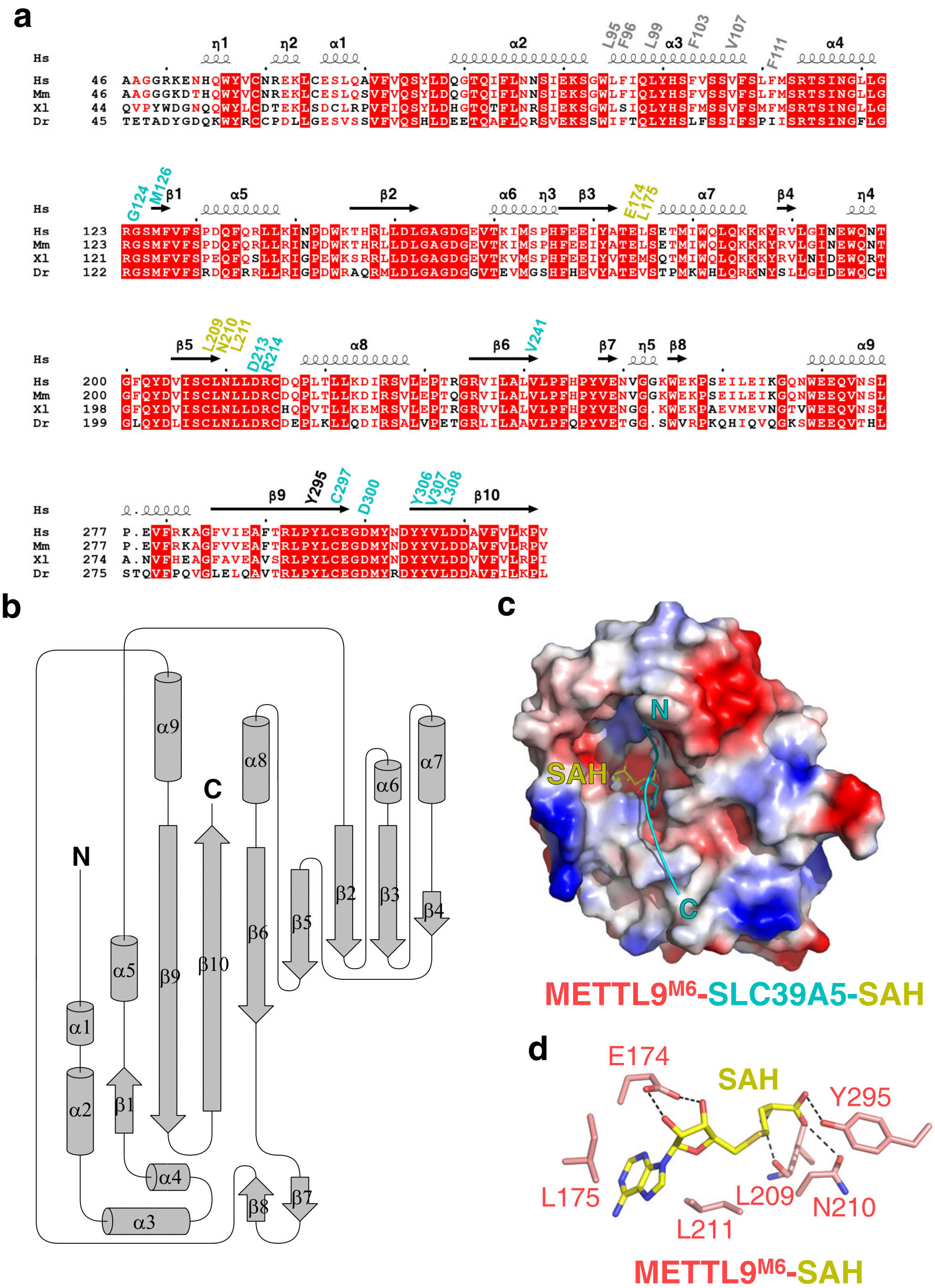

Supplementary Fig. S1. **a** Sequence alignment of METTL9 orthologs from Homo Sapiens (Hs, NP_001070648.1), Mus musculus (Mm, NP_067529.2), and Xenopus laevis (Xl, NP_001079600.1). Six METTL9 residues mutated in METTL9^{M6} are labeled in grey. METTL9 residues involved in binding to SAH and substrate histidine are shown in yellow and cyan sticks, respectively. **b** Topology diagram of METTL9^{M6} with secondary structures colored in grey. **c** Electrostatic surface of METTL9^{M6} bound with SLC39A5 (cyan cartoon) and SAH (yewllow sticks). **d** Close-up view of the interactions between SAH (yewllow sticks) and METTL9^{M6} (red sticks).

Supplementary Figure S1

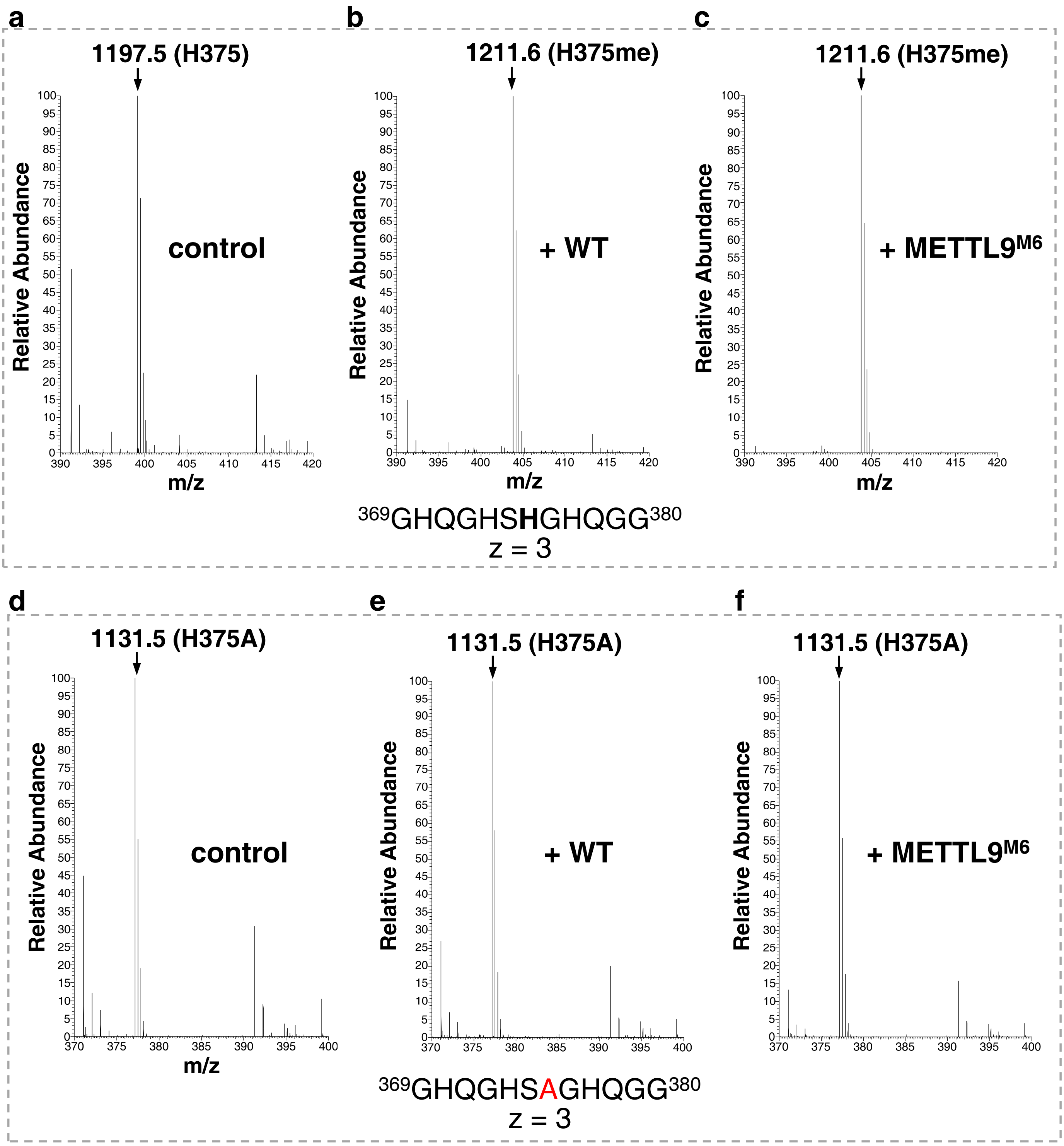

Supplementary Fig. S2. **WT METTL9 and METTL9^{M6} are active towards SLC39A5$^{369\text{-}380}$. a-c** Mass spectrometry data for SLC39A5$^{369\text{-}380}$. **a** control (no enzyme added), **b** with wild-type METTL9 (WT), **c** with METTL9^{M6}, **d-f** Mass spectrometry data for SLC39A5$^{369\text{-}380}$ H375A. **d** control (no enzyme added), **e** with wild-type METTL9 (WT), **f** with METTL9^{M6}.

Supplementary Figure S2

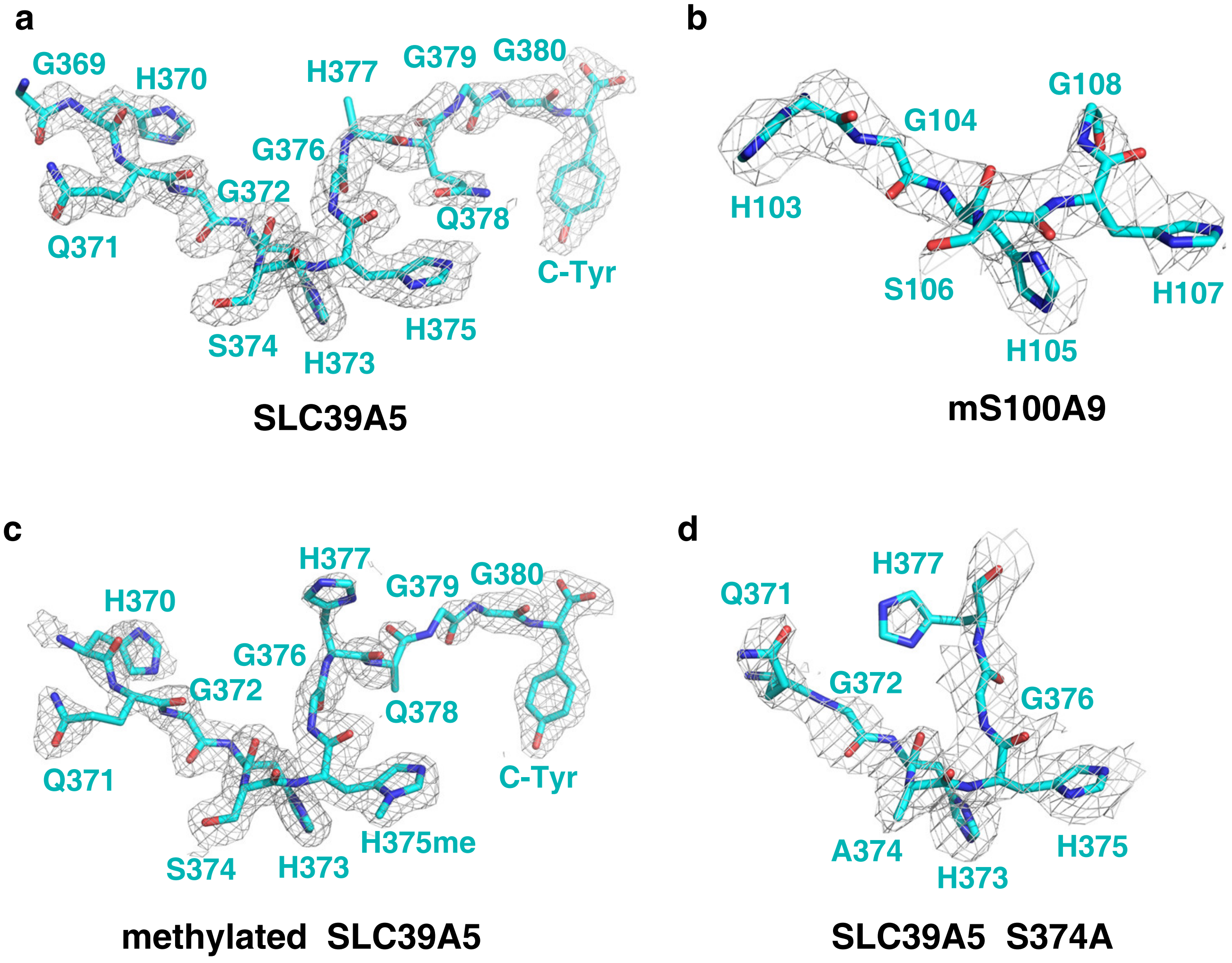

Supplementary Fig. S3. **Omit maps of peptides. a-d** The 2|Fo|–|Fc| omit maps of the peptides contoured at 1.0 σ. The peptides are shown in cyan sticks, and the maps are shown in gray meshes. **a** SLC39A5, **b** mS100A9, **c** methylated SLC39A5, and **d** SLC39A5 S374A.

Supplementary Figure S3

a

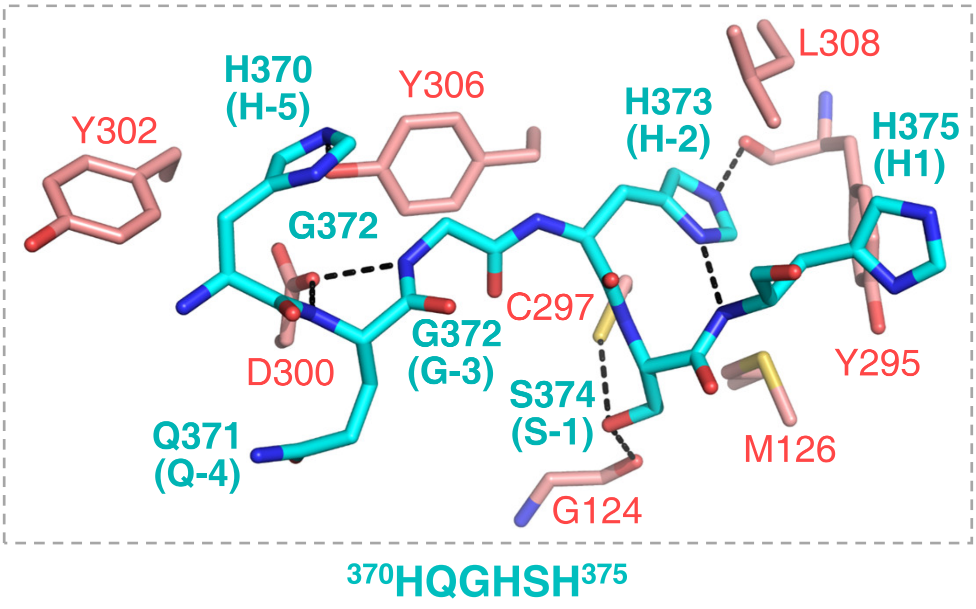

b

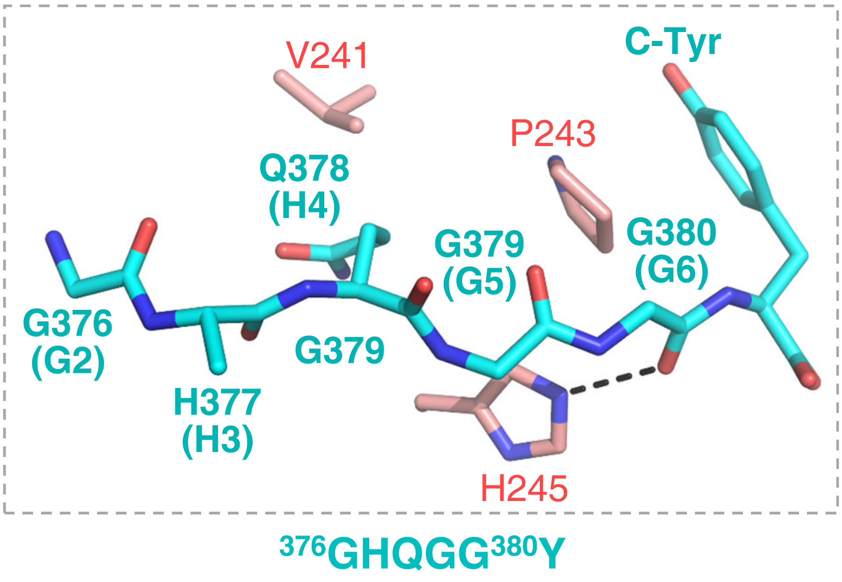

Supplementary Fig. S4. **a** Close-up view of the interactions between METTL9^{M6} (red sticks) and ^{370}HQGHSH^{375} of SLC39A5 (cyan sticks). **b** Close-up view of the interactions between METTL9^{M6} (red sticks) and ^{376}GHQGG^{380}Y of SLC39A5 (cyan sticks).

Supplementary Figure S4

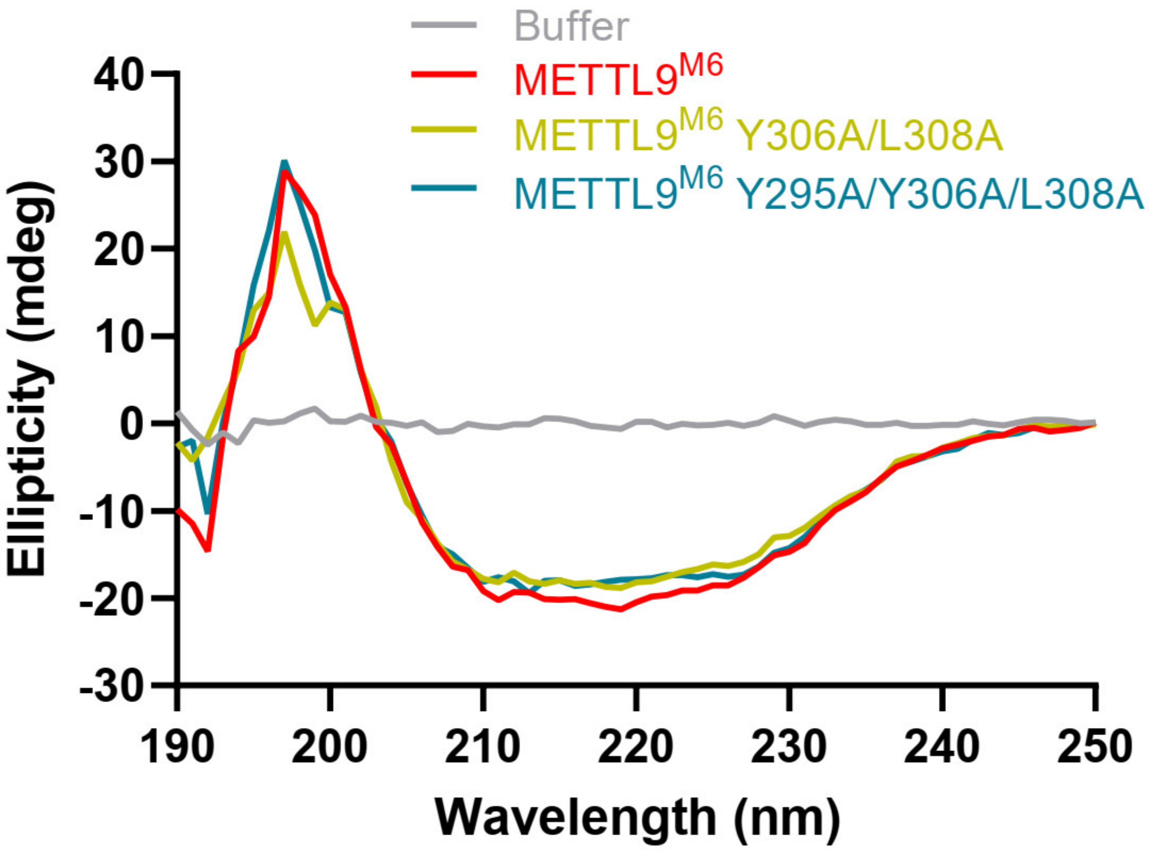

Supplementary Fig. S5. Circular dichroism (CD) spectra of METTL9^{M6} and its mutants, Y306A/L308A and Y295A/Y306A/L308A at 0.2mg/mL, with the buffer only as the control.

Supplementary Figure S5

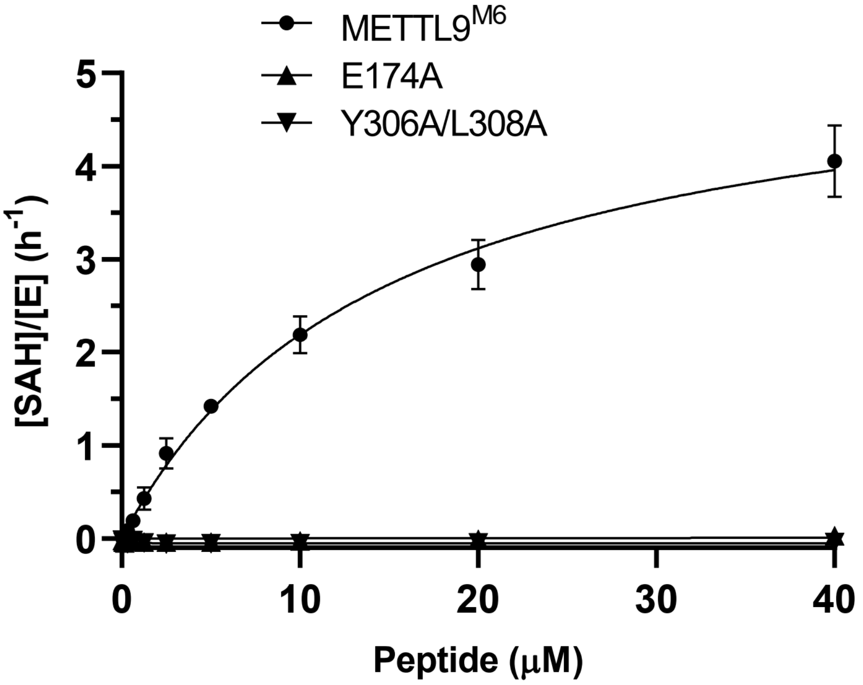

Proteins	K_m (μM)	k_{cat} (h^{-1})	k_{cat}/K_m ($\mu M^{-1} \cdot h^{-1}$)
$METTL9^{M6}$	14.7 ± 3.9	5.4 ± 0.6	0.37
E174A	*ND	ND	N/A
Y306A/Y308A	ND	ND	N/A

Supplementary Fig. S6. Steady-state kinetics of the reaction catalyzed by human $METTL9^{M6}$ and its mutants, E174A and Y306A/Y308A. The Curves for SAM at a fixed concentration of 40 μM and varying concentrations of the SLC39A5 peptide. The K_m, k_{cat}, and k_{cat}/K_m are listed in the table. ND, not detectable; N/A, not available.

Supplementary Figure S6

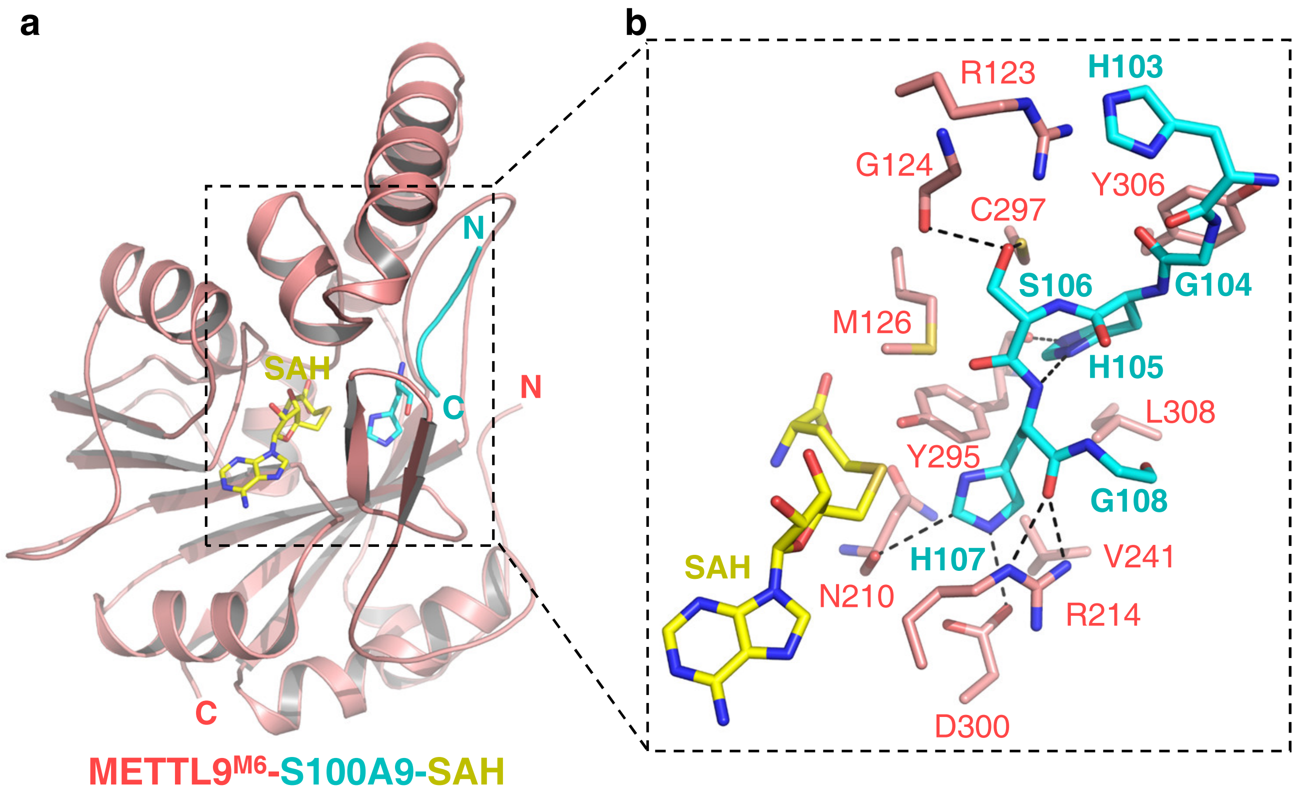

Supplementary Fig. S7. **a** Overall structure of METTL9^{M6} bound with mS100A9$^{101\text{-}111}$ and SAH, colored the same as shown in Fig. 1d. **b** Close-up view of the interactions between METTL9^{M6} and mS100A9. METTL9^{M6} residues, with residues colored in the same way as shown in Fig. 1e.

Supplementary Figure S7

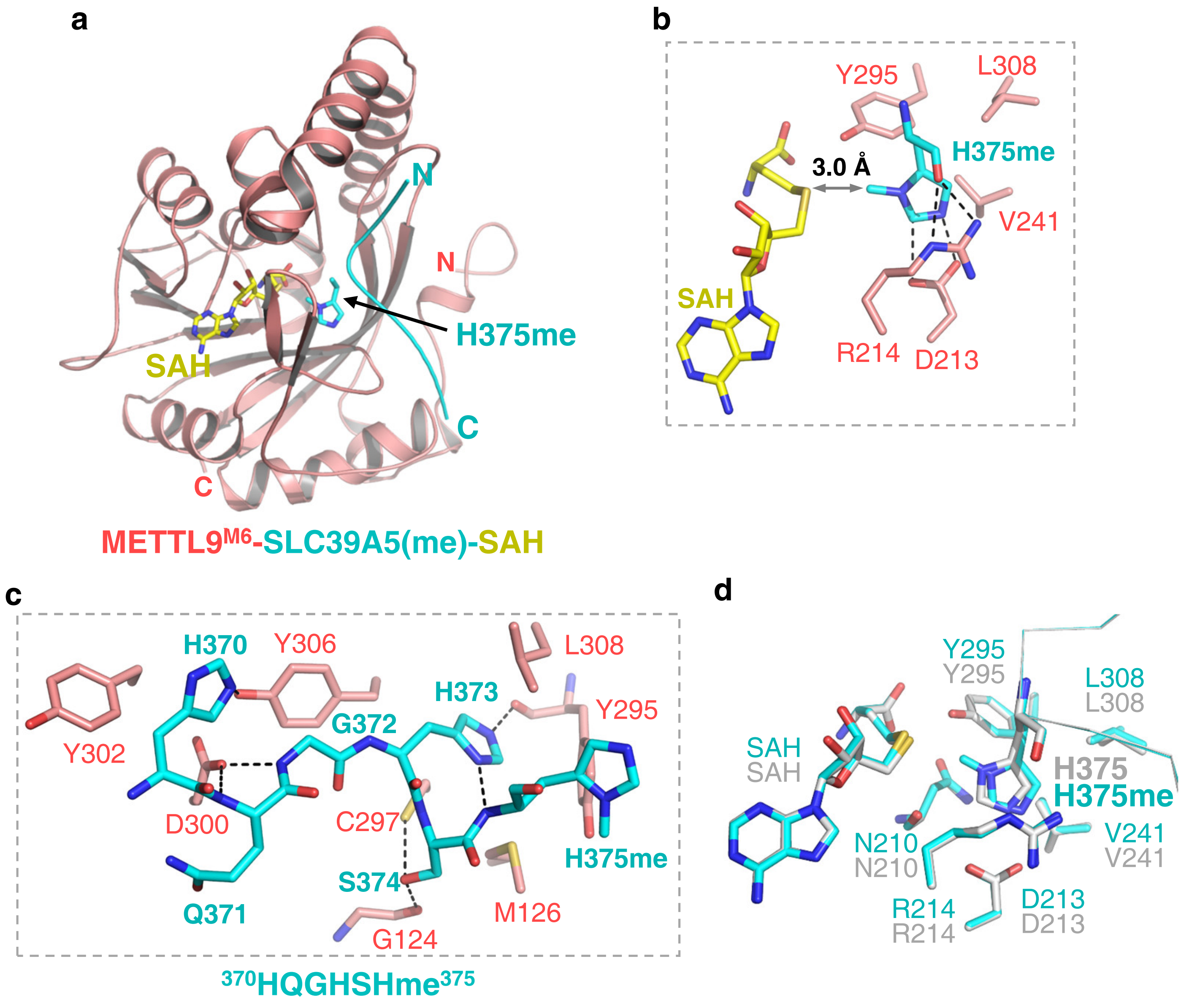

Supplementary Fig. S8. **a** Overall structure of METTL9^{M6} bound with methylated mS100A9$^{101\text{-}111}$ and SAH, which are colored the same as shown in Fig. 1d. **b** Close-up view of the interactions between METTL9^{M6} and methylated His375 of mS100A9, with residues colored in the same way as shown in Fig. 1e. The distance between SAH (yellow sticks) and methylated His375 are indicated with grey arrows. **c** Close-up view of the interactions between METTL9^{M6} and ^{370}HQGHSHme^{375}, with residues colored in the same way as shown in Fig. 1e. **d** Superposition of the SLC39A5-bound METTL9^{M6} (grey) with the methylated SLC39A5-bound METTL9^{M6} (cyan) on the histidine/methylhistidine binding pocket. The histidine/methylhistidine, histidine/methylhistidine-binding residues of METTL9^{M6} and SAH are shown in sticks.

Supplementary Figure S8

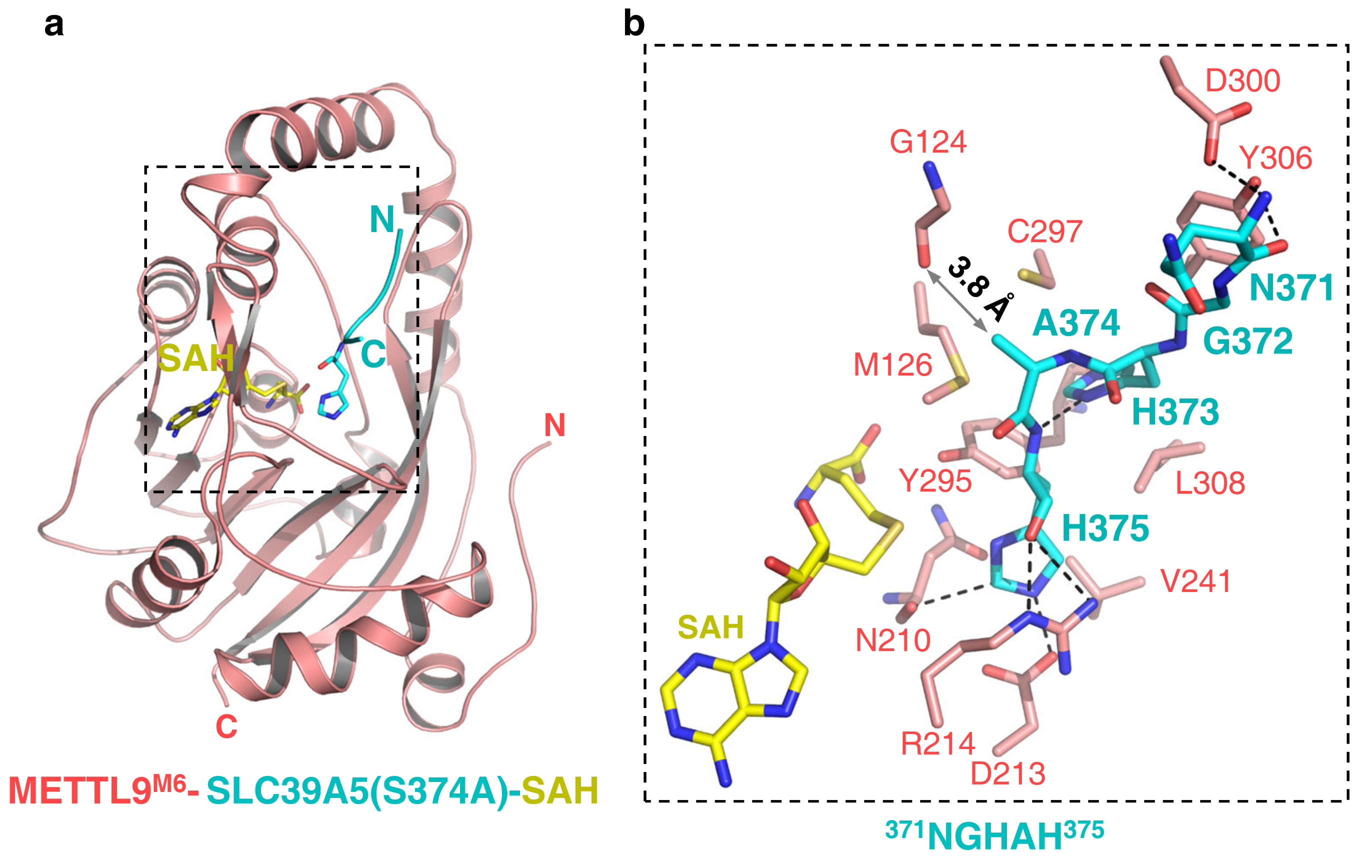

Supplementary Fig. S9. **a** Overall structure of METTL9^{M6} bound with the SLC39A5 S374A (S-1A) mutant and SAH, colored in the same manner as shown in Fig. 1d. **b** Close-up view of the interactions between METTL9^{M6} and ^{371}NGHAH^{375} of SLC39A5 S374A mutant, with residues colored in the same manner as shown in Fig. 1e. The distance between the A374 of SLC39A5 mutant and the main chain carbonyl group of METTL9^{M6} Gly124 are indicated with the grey arrow.

Supplementary Figure S9

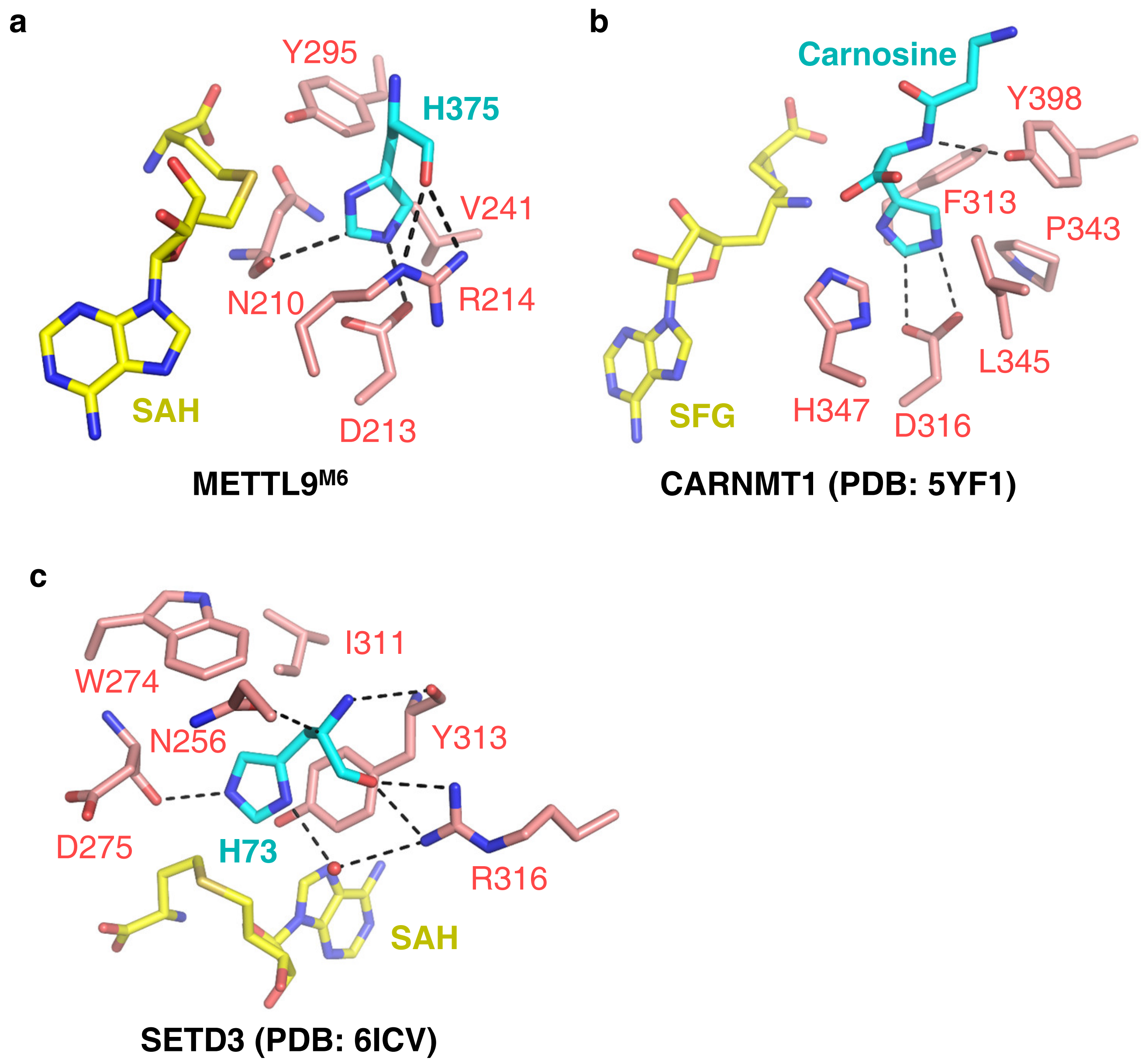

Supplementary Fig. S10. Comparing the catalytic pockets of histidine-specific methyltransferases. **a** SLC39A5 His375 is accommodated into the pocket of METTL9^{M6} in the presence of SAH. **b** Carnosine is positioned into the pocket of CARNMT1 (PDB id: 5YF1) in the presence of SINEFUNGIN (SFG). **c** Actin His73 is fitted into the pocket of SETD3 (PDB id: 6ICV) in the presence of SAH. The histidine residues, catalytic pocket residues, and SAH or SFG, are shown in cyan, red, and yellow sticks, respectively.

Supplementary Figure S10
